# Risk and attributable fraction estimation for the impact of exposure to compound drought and hot events on daily stroke admissions

**DOI:** 10.1265/ehpm.24-00168

**Published:** 2024-10-19

**Authors:** Hui Zhang, Xuezhu Li, Wenjin Shang, Tao Wu, Siyue Wang, Li Ling, Wensu Zhou

**Affiliations:** 1 School of Public Health, Sun Yat-sen University, Guangzhou, China; 2 Department of Neurology, The First Affiliated Hospital, Sun Yat-sen University; Guangdong Provincial Key Laboratory of Diagnosis and Treatment of Major Neurological Diseases; National Key Clinical Department and Key Discipline of Neurology, Guangzhou, China; 3 School of Public Health, Peking University, Beijing, China

**Keywords:** Climate change, Temperature, Compound event, Stroke, Cardiovascular health

## Abstract

**Background:**

The projection indicates that compound drought and hot events (CDHEs) will intensify, posing risks to cardiovascular health by potentially increasing stroke incidents. However, epidemiological evidence on this topic remains scarce. This study investigates the association between exposure to CDHEs and the risk of daily stroke admissions, specifically examining the effects on various stroke categories such as Subarachnoid Hemorrhage (SAH), Intracerebral Hemorrhage (ICH), Ischemic Stroke (IS), Transient Ischemic Attack (TIA), and other types of stroke.

**Methods:**

Data on daily stroke admissions from 2010 to 2015 were obtained from the Urban Employee Basic Medical Insurance (UEBMI) and Urban Resident Basic Medical Insurance (URBMI) claims databases in Guangzhou, China. Hot events were identified as days when the daily mean temperature exceeded the 75th percentile during the warm season (May to October) over the study period. The Standardized Precipitation Evapotranspiration Index (SPEI) was utilized to identify drought conditions, with thresholds set at −1 and −1.5 for low-severity and high-severity drought events, respectively. Through a generalized additive model (GAM), we analyzed the cumulative effects of CDHE exposure on daily stroke admissions and calculated the Attributable Fraction (AF) related to CDHEs.

**Results:**

The analysis included 179,963 stroke admission records. We observed a significant increase in stroke admission risks due to exposure to hot events coupled with high-severity drought conditions (RR = 1.18, 95%CI: 1.01–1.38), with IS being the most affected category (RR = 1.20, 95%CI: 1.03–1.40). The AF of total stroke admission attributed to hot events in conjunction with high-severity drought conditions was 24.40% (95%CI: 1.86%–50.20%).

**Conclusion:**

The combination of hot events with high-severity drought conditions is likely linked to an increased risk of stroke and IS admissions, which providing new insights into the impact of temperature and climate-related hazards on cardiovascular health.

**Supplementary information:**

The online version contains supplementary material available at https://doi.org/10.1265/ehpm.24-00168.

## 1. Introduction

Global warming significantly affects various societal sectors, ecosystems, and human health. The Intergovernmental Panel on Climate Change (IPCC) reports that the planet has now exceeded the critical threshold of 1.5 °C in global warming. Many world regions are becoming warmer, subjecting people to increased challenges due to hotter whether environment and extreme heat exposure. Beyond elevated temperatures, climate change models indicate that rainfall patterns will become more erratic and extreme with rising temperatures [1], thereby climate-related hazards, such as droughts, are expected to occur more frequently and with greater intensity at regional and global scales [2, 3]. These adverse weather and climate events present substantial threats to public health.

The cardiovascular system is particularly vulnerable to the effects of a changing climate, including specific diseases like stroke [4, 5]. Stroke is a condition characterized by high mortality and disability rates, as well as a significant risk of recurrence, but which is also a preventable disorder [6]. A growing body of epidemiological evidence identifies hot whether (including high temperatures and extreme heat whether) as a novel risk factor for stroke, elevating the likelihood of hospital admissions, emergency department visits, and outpatient visits related to stroke [7–10]. The biological mechanism for the relationship between high temperature and stroke has also been explained [11]. As for drought events, previous research has underscored their independent impact on cardiovascular outcomes [12, 13], although findings have been inconsistent. For example, a study in the Northern Rockies and Plains of the United States found a correlation between drought conditions and increased cardiovascular mortality [12]. Similarly, evidence from counties in the western U.S. showed a positive relationship between drought exposure and cardiovascular admissions [14]. Conversely, another study found no significant difference in cardiovascular admissions during drought conditions [13]. These investigations suggest a potential link between drought and stroke.

However, climate variables are interrelated. Hot and drought often occur simultaneously, referred to as compound drought and hot events (CDHEs) [15]. Warmer temperatures accelerate evaporation, altering water availability timing and leading to drier warm seasons. Unlike their independent effects, these compound events can amplify health risks beyond those posed by single factors, leading to more significant health and well-being implications [16]. For stroke, the biological mechanism suggests that simultaneous exposure to high temperature and drought can increase blood viscosity and affect metabolism [17]. Additionally, drought conditions, coupled with excessive sweating and increased water evaporation on hot days [18], make recovery from single environmental exposures more challenging. Thus, understanding the relationship between compound hot-drought events and stroke is critical. To date, no studies have quantified the impacts of such compound events on stroke. Identifying population vulnerabilities is also beneficial, as individual characteristics may influence the observed environmental risks-health relationship in current studies and help design targeted mitigation strategies to sub-populations [19]. Stroke, known for its high mortality, disability rates, and significant recurrence risk, requires prompt and timely treatment [20]. This represents a critical gap in stroke prevention, which calls for a deeper understanding of the association between CDHEs and stroke considering the future challenges posed by climate change.

Therefore, the present study aims to explore the hypothesis that CDHEs increase stroke risks, influencing hospital admissions for stroke its specific categories and also affecting various stroke categories differently across population segments, such as by gender and age. By filling this research gap, our study attempts to provide valuable insights into the intricate relationship between climate change, extreme weather events, and cardiovascular health outcomes.

## 2. Methods

### 2.1. Study site

This study was conducted in Guangzhou, China, the capital of Guangdong Province and one of the most economically developed cities in Southern China (Fig. S1). As of the end of 2022, Guangzhou had a permanent population of 18,734,100, with an urbanization rate of 86.48%. The city’s gross domestic product (GDP) stood at 288.39 billion yuan. Geographically, Guangzhou is situated at 23°117′ north latitude and 113°276′ east longitude. It experiences a subtropical monsoon climate characterized by warm summers and mild winters. The average annual temperature in Guangzhou is 21.0 °C, with July being the hottest month, boasting an average monthly temperature of 28.7 °C. The warm season extends from May to October [21]. Guangzhou is one of the cities particularly vulnerable to climate-related health risks, such as extreme heat and drought events, in China [22].

### 2.2. Data collection

Daily hospital admissions over the study period from 2010 to 2015 were obtained from the Urban Employee Basic Medical Insurance (UEBMI) and the Urban Resident Basic Medical Insurance (URBMI) claims databases of Guangzhou city, which recorded the gender, age, as well as the type of hospital visits and the number of daily hospital visits. The UEBMI and URBMI were two common basic medical insurance scheme in China and has highly cover rate. For instance, in China, the two insurance schemes have together covered 78% of the Chinese population in 2011 [23, 24], thus, the dataset has a good representation for the whole population. We obtained all the reimbursement claims submitted for hospitalization with the single primary diagnosis of stroke from 2010 to 2015 using the International Classification of Diseases (ICD) Codes tenth version. All of cases we used have already been diagnosed as stroke by health professionals, which included subarachnoid haemorrhage (SAH) (ICD-10: I60), intracerebral haemorrhage (ICH) (ICD-10: I61), ischaemic stroke (IS) (ICD-10: I63, I66), transient ischaemic attack (TIA) (ICD-10: G45) and other types of stroke (ICD-10: I64, I67–I69). In our study, we excluded all records containing information about patients under 18 years old.

We collected daily meteorological data including daily mean temperature (Tmean, °C), atmosphere pressure and relative humidity (RHmean, %) in Guangzhou from 2010 to 2015 from the Guangzhou Meteorological Bureau. Given the availability of data, our analysis accounts for only two air pollutants as confounding factors. Contemporaneous daily mean concentrations of ambient air pollutants (i.e., PM_2.5_ and O_3_) in Guangzhou were obtained from China High Air Pollutants (CHAP) database [25, 26]. Data on PM_2.5_ and O_3_ has high quality, as PM_2.5_ as an example, China High PM_2.5_ is one of the series of long-term, full-coverage, high-resolution, and high-quality datasets of ground-level air pollutants for China and yields a high quality with a cross-validation coefficient of determination (CV-R^2^) of 0.92, a root-mean-square error (RMSE) of 10.76 µg/m^3^, and a mean absolute error (MAE) of 6.32 µg/m^3^ on a daily basis.

### 2.3. Definition for CDHEs

We defined a hot day event was based on the daily mean temperature information. Previous studies have confirmed a hot event as when the daily mean temperature exceeds its 75th percentile during the warm season over the entire study period [27]. It is also helpful to obtain enough hot events in the analysis. To measure drought exposure, we used the daily Standardized Precipitation Evapotranspiration Index (SPEI), a commonly employed index to reflect drought conditions. The daily SPEI data, with a spatial resolution of 0.1° × 0.1°, was sourced from a dataset developed by Zhang and colleagues [28]. The daily SPEI data were extracted by calculating statistics on the cell values of a raster within the zones covered by Guangzhou city. Additionally, daily temperature data were collected from the Guangzhou Meteorological Bureau, which provided daily temperature records for the city. Previous research has indicated that the SPEI is more sensitive in identifying drought conditions linked to warmer months and extreme heat [29]. Our research mainly focused on short-term drought event, thereby the SPEI operates on a 30-day accumulation scale was used to reflect drought conditions. For identifying drought and wet events, thresholds of −1 and 1 were used, respectively, with values beyond −1.5 or above 1.5 indicating more severe drought or wet conditions [18, 30, 31].

**Fig. 1:**
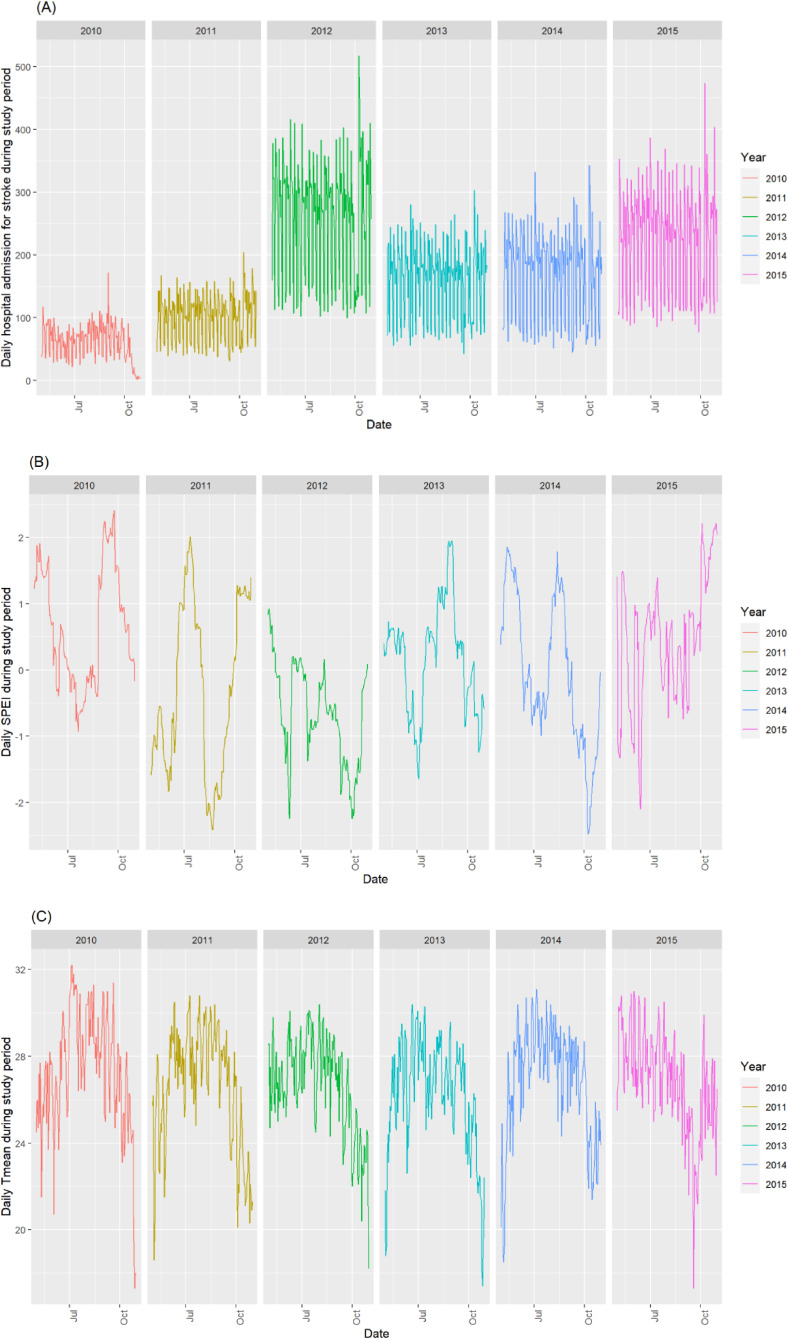
Decomposition of daily admission for stroke (A), SPEI (B) and Tmean (C) during study period

Accordingly, we classified the study period into six categories based on drought and high temperature thresholds: 1) an independent hot event, characterized by a daily mean temperature above the 75th percentile with SPEI within the normal range (−1 < SPEI < 1); 2) hot event & low-severity drought (SPEI between −1.5 and −1); 3) hot event & high-severity drought (SPEI < −1.5); 4) hot event & low-severity wet conditions (SPEI between 1 and 1.5); 5) hot event & high-severity wet conditions (SPEI > 1.5); and 6) days with normal temperatures. Consequently, we treated the variable for the compound hot-drought event as a categorical factor. Days with normal temperatures were used as the reference category, defined by any remaining days that had normal temperatures, regardless of the SPEI values. This categorization allows for the differentiation of drought intensity levels and accounts for a sufficient number of days under these conditions during the study period. Decomposition of daily admission for stroke, SPEI and Tmean during study period were shown in Fig. 1.

### 2.4. Statistical analysis

This is a time-series analysis in which we quantify the association between CDHEs and daily admissions for stroke by employing a quasi-Poisson generalized additive model (GAM): This formula represent as:

$$\begin{array}{rl} \mathrm{Log}[E(Y_{\text{t}})] & =\alpha +\text{cb}({\text{CDHEs}}_{t})+\text{ns}(\text{time},4/\text{year})\\ & \;+\text{ns}(\text{relative humidity},3)\\ & \;+\text{ns}(\text{atmosphere pressure},3)\\ & \;+\text{ns}({\text{PM}}_{2.5},3)+\text{DOW} \end{array}$$

In this analysis, t represents the day of observation; E(Yt) denotes the counts of daily admissions for stroke on day t; the log function serves as the link function; α was the model intercept; CDHEs_t_ is a categorical variable indicating the presence of a hot event, a hot event combined with low-severity drought, a hot event combined with high-severity drought, a hot event combined with low-severity wet conditions, and a hot event combined with high-severity wet conditions. Focusing on hot and drought events, normal temperature days were designated as the reference group, and we primarily reported the cumulative effects of a hot event, a hot event combined with low-severity drought, and a hot event combined with high-severity drought. These were included and fitted with a distributed lag non-linear model (DLNM), which employs an integer function and a natural cubic spline function with 2 degrees of freedom (dfs) to calculate the delayed effects of compound drought and hot events, respectively [27]. Considering the acute and short-term effect of compound events, we utilized a maximum lag period of 3 days to capture the lagged effect of compound events on daily admissions for stroke. Additionally, this model accounts for atmospheric pressure and relative humidity, which were modeled as spline functions with 3 degrees of freedom (dfs) to control for their potential confounding effects. PM_2.5_ was included also by a spline function with 3 df. Long-term time trend and seasonal trend were also considered. For instance, dow is denotes a categorical variable of day of week and the time indicator with 4 dfs for each year also be included [32]. In the analysis, total admissions for stroke, and its five specific categories were set as dependent variables respectively. All of estimations were presented by the relative ratios (RR) and 95% confidence interval (CI). The main findings were reported by showing the cumulative effect of compound events relative to reference normal day.

Identifying population vulnerabilities is our second aims, we conducted subgroup analysis by repeated analysis in gender (Male, Female) and age (<60 years, ≥60 years). In addition to RR estimations, public health researchers also computed attributable fraction (AF) and attributable deaths of environmental factor, which provide more information for and public health implication [33, 34]. In this study, AFs of hot event, hot event combined with low-severity drought, hot event combined with high-severity drought were calculated. This was done by dividing the total number of admissions attributable to each of these conditions by the total number of admissions for their respective categories. The 95% confidence interval (CI) of AF is calculated using Monte Carlo simulations by simulating 1000 random samples. The 95%CI can be extracted as the percentiles related to the 2.5th and 97.5th of simulated distributions. The differences among gender and age subgroups were assessed by calculating the 95% CI using the following formula as: $(b_{1}-b_{2})\pm 1.96\sqrt{({\mathrm{SE}}_{1}{)}^{2}+({\mathrm{SE}}_{2}{)}^{2}}$ , were b_1_ and b_2_ represent the point estimates for each group, and SE_1_ and SE_2_ denote the standard errors [35].

Sensitivity analyses were conducted to confirm the robustness of our findings. First, we changed the maximum lag periods from 3 days to 1 day and 2 days to determine if our findings remained consistent across different lag durations. Second, we excluded PM_2.5_ from the model to assess the effects in the absence of air pollution factor. Concurrently, the main model was refitted to include both PM_2.5_ and O_3_. Third, we also excluded humidity to examine the underlying effect attributed by humility. Fourth, we modified the categorization of hot-drought events to investigate if the definitions for the division of hot and drought events influence the model’s output. Initially, hot-drought events were categorized into four groups: normal temperature, hot event, hot event combined with low-severity drought, and hot event combined with high-severity drought. This was expanded to eight categories: normal temperature & neither wet nor drought conditions, independent wet conditions with normal temperature but SPEI > 1, independent drought conditions with normal temperature but SPEI < −1, hot event, hot event with low-severity wet, hot event with high-severity wet, hot event with low-severity drought, and hot event with high-severity drought.

All statistical analyses were conducted were conducted with R software (version 4.2.2). A two-tailed P-value below 0.05 was deemed to indicate statistical significance.

## 3. Results

### 3.1. Basic descriptive results

During 2010 to 2015, a total of 179,963 records of hospital admissions for stroke were obtained, including 1,985 for SAH, 107,661 for IS, 16,562 for ICH, 5,051 for TIA, and 48,704 for OTS (Table 1). The proportions of hot events, hot events with low-severity droughts, and hot events with high-severity droughts accounted for 15.6%, 2.3%, and 2.2% over the entire period. According to Table 2, during the hot events, hot events with low-severity droughts, and hot events with high-severity droughts, the mean Tmean temperatures were 29.7 ± 0.87 °C, 29.8 ± 0.64 °C, and 29.4 ± 0.60 °C, respectively. The corresponding SPEIs were −0.14 ± 0.52, −1.20 ± 0.14, and −1.97 ± 0.24, respectively.

**Table 1:** Descriptive statistics of hospital admission for stroke stratified by hot and drought events

**Variables**	**All**		**Normal temperature**		**Hot event**		**Hot event & low-severity drought**		**Hot event & high-severity drought**		**Hot event & low-severity wet**		**Hot event & high-severity wet**	
						
	**Total**	**Mean ± SD**	**Total**	**Mean ± SD**	**Total**	**Mean ± SD**	**Total**	**Mean ± SD**	**Total**	**Mean ± SD**	**Total**	**Mean ± SD**	**Total**	**Mean ± SD**
Daily mean admission (cases)	179,963	163 ± 94.3	135,778	165 ± 95.3	28,705	166 ± 96.5	4,827	186 ± 94.8	3,178	127 ± 75.3	5,084	154 ± 77.6	2,391	104 ± 43.6
Age group (year)														
<60	33,410	30.3 ± 18.7	25,311	30.7 ± 18.8	5,243	30.3 ± 19.6	932	35.8 ± 19.1	566	22.6 ± 15.4	942	28.5 ± 15.8	416	18.1 ± 8.64
≥60	146,553	133 ± 76.8	110,467	134 ± 77.8	23,462	136 ± 77.9	3,895	150 ± 76.5	2,612	104 ± 60.8	4,142	126 ± 63.5	1,975	85.9 ± 36.5

Sex														
Male	94,206	85.3 ± 49.4	71,193	86.4 ± 49.9	14,889	86.1 ± 50.3	2,510	96.5 ± 50.3	1,708	68.3 ± 40.8	2,674	81.0 ± 41.9	1,232	53.6 ± 23.8
Female	85,757	77.7 ± 46.1	64,585	78.4 ± 46.6	13,816	79.9 ± 47.5	2,317	89.1 ± 45.4	1,470	58.8 ± 35.3	2,410	73.0 ± 37.1	1,159	50.4 ± 21.0
Types of stroke														
IS	107,661	97.5 ± 54.9	80,795	98.1 ± 55.5	17,393	101 ± 57.2	2,832	109 ± 53.7	2,038	81.5 ± 46.3	3,071	93.1 ± 45.5	1,532	66.6 ± 22.3
SAH	1,985	1.80 ± 1.77	1,496	1.80 ± 1.77	335	1.94 ± 2.02	50	1.92 ± 1.81	34	1.36 ± 1.29	46	1.39 ± 1.20	24	1.04 ± 1.11
ICH	16,562	15.0 ± 9.18	12,642	30.7 ± 18.8	2,543	30.3 ± 19.6	426	35.8 ± 19.1	308	22.6 ± 15.4	410	28.5 ± 15.8	233	18.1 ± 8.64
TIA	5,051	4.58 ± 5.41	3,849	4.67 ± 5.44	767	4.43 ± 5.87	129	4.96 ± 5.85	141	5.64 ± 3.89	69	2.09 ± 3.30	96	4.17 ± 3.28
OTS	48,704	44.1 ± 32.3	36,996	44.9 ± 32.7	7,667	44.3 ± 31.6	1,390	53.5 ± 34.1	657	26.3 ± 21.0	1,488	45.1 ± 29.6	506	22.0 ± 18.2

**Table 2:** Descriptive statistics daily meteorological factors and air pollutants during study period

**Variables**	**Normal temperature**	**Hot event**	**Hot event & low-severity drought**	**Hot event & high-severity drought**	**Hot event & low-severity wet**	**Hot event & high-severity wet**
					
	**Mean ± SD**	**Mean ± SD**	**Mean ± SD**	**Mean ± SD**	**Mean ± SD**	**Mean ± SD**
Daily mean temperature (°C)	25.8 ± 2.23	29.7 ± 0.87	29.8 ± 0.64	29.4 ± 0.60	29.4 ± 0.66	29.6 ± 0.83
Daily SPEI	0.09 ± 1.07	−0.14 ± 0.52	−1.20 ± 0.14	−1.97 ± 0.24	1.28 ± 0.16	1.91 ± 0.21
Daily relative humidity (%)	0.81 ± 0.10	0.74 ± 0.06	0.75 ± 0.03	0.75 ± 0.05	0.74 ± 0.08	0.76 ± 0.06
Daily atmospheric pressure (nPa)	1000 ± 4.82	998 ± 4.01	998 ± 2.56	998 ± 3.54	999 ± 2.91	1000 ± 4.20
Daily PM_2.5_ (µg/m^3^)	39.1 ± 18.3	31.9 ± 13.8	26.0 ± 9.56	34.3 ± 14.8	30.7 ± 11.5	38.7 ± 9.88
Daily O_3_ (µg/m^3^)	98.9 ± 34.4	121 ± 28.5	103 ± 22.0	116 ± 23.2	116 ± 27.2	123 ± 25.7

### 3.2. Risk and AF estimation for the impact of exposure to CDHEs on daily stroke admissions

Table 3 lists the cumulative effects of CDHEs on the risks of daily stroke admissions. As we could see, a hot event combined with high-severity drought significantly increased the risk of daily stroke admissions (RR = 1.18, 95%CI: 1.01–1.38). However, we did not find any significant association between an independent hot event (RR = 1.05, 95%CI: 0.97–1.12) and or a hot event combined with low-severity drought (RR = 1.00, 95%CI: 0.88–1.14) and daily admissions for stroke.

**Table 3:** Cumulative risks of stroke from different hot events by sex, age and category for stroke.

	**Hot event**			**Hot event & low-severity drought**			**Hot event & high-severity drought**		
		
	**RR**	**95%CI**		**RR**	**95%CI**		**RR**	**95%CI**	
All	1.05	0.97	1.12	1.00	0.88	1.14	**1.18**	**1.01**	**1.38**
Types of stroke									
SAH	0.98	0.77	1.25	0.71	0.45	1.10	0.97	0.59	1.59
IS	1.05	0.97	1.13	1.00	0.88	1.15	**1.20**	**1.03**	**1.40**
ICH	1.04	0.93	1.17	1.12	0.92	1.37	1.16	0.91	1.47
TIA	1.12	0.93	1.34	1.08	0.78	1.50	1.07	0.79	1.44
OTS	1.03	0.95	1.12	0.97	0.84	1.13	1.17	0.94	1.45
Age									
Age ≥60	1.05	0.98	1.13	0.98	0.86	1.12	**1.19**	**1.01**	**1.39** ^a^
Age <60	1.02	0.94	1.12	1.09	0.93	1.27	1.16	0.95	1.41
Gender									
Male	1.04	0.96	1.12	1.00	0.87	1.14	**1.17**	**1.00**	**1.38** ^b^
Female	1.06	0.98	1.14	1.01	0.87	1.16	**1.19**	**1.00**	**1.41**

The bold values mean statistical significance.

^a^*P* for the result of difference between age groups = 0.85.

^b^*P* for the result of difference between gender groups = 0.89.

According to Table 4, which shows the AFs by compound drought and hot events, the estimation was 23.40% (95%CI: 1.86%–50.20%). As for hot events and hot events combined with low-severity drought, AFs were 4.52% (95%CI: −2.93%–12.60%) and 0.10% (95%CI: −9.93%–12.31%), respectively. Regarding specific types of stroke, IS is predominantly affected by a hot event combined with high-severity drought (RR = 1.20, 95%CI: 1.03–1.40, AF = 24.13%, 95%CI: 4.33%–47.85%).

**Table 4:** Attributable fraction (%) from different hot events by sex, age and category for stroke.

	**Hot event**			**Hot event & low-severity drought**			**Hot event & high-severity drought**		
		
	**AF**	**95%CI**		**AF**	**95%CI**		**AF**	**95%CI**	
All	4.52	−2.93	12.60	0.10	−9.93	12.31	23.40	1.86	50.20
Types of stroke									
SAH	−1.55	−21.95	23.43	−27.80	−50.83	7.35	−4.34	−55.01	87.31
IS	4.86	−2.65	12.74	0.41	−10.77	13.61	24.13	4.33	47.85
ICH	4.12	−8.40	16.52	11.43	−6.35	34.83	19.49	−11.85	55.76
TIA	12.19	−7.36	34.83	7.25	−21.80	45.06	5.39	−17.94	39.58
OTS	3.33	−5.49	13.44	−2.26	−13.11	11.43	28.59	−9.30	72.27
Age									
Age ≥60	4.96	−2.18	13.21	−1.64	−12.01	11.22	23.85	1.13	48.62
Age <60	2.49	−6.90	12.58	7.56	−5.97	22.94	21.15	−9.48	51.59
Gender									
Male	3.56	−3.11	11.23	−0.26	−10.81	12.17	22.07	0.84	47.20
Female	5.57	−2.27	14.20	0.49	−10.20	14.10	24.98	0.29	55.22

### 3.3. Subgroup analysis

Table 3 and Table 4 also present findings from subgroup analyses for risk and AFs estimation. The effects of compound events were significant among older adults (RR = 1.19, 95%CI: 1.01–1.39, AF = 23.85%, 95%CI: 1.13%–48.62%), females (RR = 1.19, 95%CI: 1.00–1.41, AF = 24.98%, 95%CI: 0.29%–55.22%), and males (RR = 1.17, 95%CI: 1.00–1.38, AF = 22.07%, 95%CI: 0.84%–47.20%). However, we did not find estimate differences for RRs between younger and older participants (*P* Value for effect modification was 0.85), nor between male and female participants (*P* Value for effect modification was 0.89) (Table S4).

### 3.4. Sensitivity analysis

In our sensitivity analysis, we observed that altering the maximum lag period or the df for the time variable did not affect our findings, our results consistently showed a positive relationship between hot events combined with high-severity drought and daily stroke admissions (Table S1). Similarly, our findings remained unchanged when we excluded PM_2.5_ from the analysis and when we simultaneously included PM_2.5_ and O_3_ (Table S2). Furthermore, modifying the categorization of hot-drought events did not impact the significant association between hot events combined with high-severity drought and the increased risks of daily stroke admissions, indicating that changes to the definition did not affect the main findings (Table S3). Therefore, none of the sensitivity analyses materially altered our main conclusions.

## 4. Discussion

Enhancing the understanding of the link between CDHEs and stroke, is imperative for assessing climate-related risks. To our knowledge, this study is one of the few that explores this topic by focusing on compounded events, hospital admissions for stroke, and examining effect modifiers. We discover that CDHEs, particularly when hot events combined with severe droughts, significantly increase the risk of stroke hospitalizations, with IS being the most affected.

At present, published studies have reported a significant link between high temperatures and the risk of stroke. For example, Zhou et al. [36] found that the heat-related mortality risk for stroke was 1.54 (95% CI: 1.44–1.65). Another study indicated that extreme heat increased the risk of stroke occurrence in China [37]. A systematic review indicated a stroke morbidity risk associated with high ambient temperatures of 1.01 (95% CI: 1.02–1.08) [10]. Limited evidence has indicated a correlation between drought events and an increase in cardiovascular mortality. Two surveys conducted early in Spain reported statistically significant correlations between periods of drought and daily mortality rates related to circulatory systems [13, 38]. A recent published survey suggested that drought conditions could lead to approximately a 5% rise in deaths related to cardiovascular issues [12]. However, focusing solely on individual climate variables might not fully capture the comprehensive impact of extreme weather and climate hazards, particularly in the context of changing climate conditions [22]. Our research found an increased risk of stroke admissions during periods characterized by both high temperatures and severe drought conditions. However, lack of studies exploring the impact of combined drought and heat events on stroke incidence constrains our ability to draw comparisons between our results and existing research. Another new study, which analyzed mortality data from 353 locations in China, examined the combined effects of temperature and humidity on mortality. It found that high temperatures coupled with low relative humidity levels increased all-cause mortality risk, with the greatest risk observed during simultaneous high-temperature and low-humidity events [27]. While our findings align with those presented by Fang et al., highlighting the detrimental effects of the dry-hot combination, our study distinguishes itself by employing a drought index, which offers a more comprehensive perspective of current conditions and better reflects the relative long-term cumulative impacts (e.g., 30-day scale in our study) of drought compared to single meteorological indicators. This study also assessed the contribution of CDHEs to the hospital admission burden for stroke, offering valuable insights for health policy. That is, approximately 20% of risks could be prevented by considering the role of environmental factors, specifically by avoiding exposure to high temperatures and severe drought conditions. This is of significant concern, given the high global incidence of new stroke cases and related deaths [39], alongside the challenges posed by global warming, the rapid pace of urbanization, and the aging population. Furthermore, our study revealed that admissions for IS were notably impacted, with larger effect estimates and AFs than those observed for other stroke types admissions. IS, an urgent medical condition caused by reduced blood flow to the brain leading to brain cell damage, demands immediate recognition and emergency treatment [40]. While the mechanisms explaining the link between high ambient temperature combined with drought and IS risk remain unclear, some literature offers potential explanations. Previous studies indicated exposure to high temperatures prompts the body to enhance heat dissipation through thermoregulatory responses, such as sweating, which can lead to dehydration [17]. Meanwhile, drought conditions can lead to increased water evaporation from the body, resulting in diminished blood flow [11, 41]. This reduction can decrease the blood supply to the brain, potentially exacerbating existing ischemic conditions [42], particularly in individuals with severe stenosis or occlusion of large blood vessels. Furthermore, dehydration is associated with hypercoagulable states, which can induce clot formation and lead to ischemic stroke events [43]. However, further research is needed to elucidate the mechanisms behind the significant relationship between combined heat and drought exposure and IS risk.

Our subgroup analysis revealed that the relationship between combined heat and drought events and hospitalization risks was significant among older adults, males, and females. However, the effect estimates between categories within age and gender subgroups were not significant. Therefore, we believe that the interpretation of our subgroup analysis should be applied with caution, and need to be considered in future studies.

This study attempts to examine the effects of compound hot and drought events on hospitalizations due to stroke using basic medical insurance database. Meanwhile, we take into account varying levels of drought severity, types of strokes, and demographic differences based on gender and age, all of which hold significant implications for public health. However, there are several limitations that should be acknowledged. Firstly, this was a cross-sectional study that utilized a time-series ecological design to investigate the combined effects of heat and drought on stroke risk. This approach has low fidelity and is susceptible to ecological fallacy, thereby no causal relationships can be established. Second, our study sample originated from a single large city, distinguished by its developed economy and high level of urbanization. This restriction may result in measurement errors when assessing exposure levels. Consequently, caution is required when generalizing our results to other locations. Thirdly, in the present study, five types of stroke - SAH, ICH, IS, TIA and other types of stroke were examined. Due to data limitations, we did not have access to the subtypes of IS. Future studies could consider further analysis of the subtypes of ischemic stroke, including atherosclerotic, lacunar, and cardiogenic strokes, and explore the possible different mechanisms involved. Fourth, our primary emphasis was on the short-term effects of drought events; however, the long-term impacts warrant further investigation in future studies. Lastly, recent years have projected a rise in the frequency, intensity, and duration of hot events. It is imperative to utilize current data to validate the findings of our study.

## 5. Conclusion

In conclusion, this study highlights the evidence of the risks associated with the simultaneous occurrence of drought and hot weather events on daily stroke admissions. Our findings reveal that such combined conditions lead to an 18% increase in total stroke admissions and a 20% increase in IS admissions, with AFs of 23.40% and 24.13%, respectively. These findings highlight the critical role of early warning systems and the need for collaborative efforts among various departments and organizations. Such initiatives are essential for mitigating the adverse impacts and reducing the overall burden associated with climate change-related hazards.

## Supplementary information

**Additional file 1:** **Figure S1.** The location of Guangzhou city in present study. **Table S1.** Cumulative relative risks of hospital admission for stroke from different hot events by sex, age and categorical for stroke by adjusting the maximum lag periods and removing humility. **Table S2.** Cumulative relative risks of hospital admission for stroke from different hot events by sex, age and categorical for stroke by adjusting the air pollution. **Table S3.** Cumulative relative risks of hospital admission for stroke from different hot events by sex, age and categorical for stroke by adjusting the categories of events. **Table S4.** Cumulative risks of stroke from different hot events by sex and age.

## Declarations

### Availability of data and material

Data sharing not applicable to this article as no datasets were generated or analyzed during the current study.
