# Supplementary material for: Risk and attributable fraction estimation for the impact of exposure to compound drought and hot events on daily stroke admissions
Source: Environ Health Prev Med. 2024 Oct 19;29:56. doi: 10.1265/ehpm.24-00168 (PMC11524747; doi:10.1265/ehpm.24-00168)
Supplement: Supplementary file 1 — Additional file 1: Figure S1. The location of Guangzhou city in present study. Table S1. Cumulative relative risks of hospital admission for stroke from different hot events by sex, age and categorical for stroke by adjusting the maximum lag periods and removing humility. Table S2. Cumulative relative risks of hospital admission for stroke from different hot events by sex, age and categorical for stroke by adjusting the air pollution. Table S3. Cumulative relative risks of hospital admission for stroke from different hot events by sex, age and categorical for stroke by adjusting the categories of events. Table S4. Cumulative risks of stroke from different hot events by sex and age. [file ehpm-29-056-s001.docx]

**Supplementary Materials to**

**Risk and attributable fraction estimation for the impact of exposure to compound drought and hot events on daily stroke admissions**

**Table of contents**

| Table/Figure | Contents | Page |
| --- | --- | --- |
| **Figure S1.** | The location of Guangzhou city in present study | 2 |
| **Table S1.** | Cumulative relative risks of hospital admission for stroke from different hot events by sex, age and categorical for stroke by adjusting the maximum lag periods and removing humility | 3 |
| **Table S2.** | Cumulative relative risks of hospital admission for stroke from different hot events by sex, age and categorical for stroke by adjusting the air pollution | 4 |
| **Table S3.** | Cumulative relative risks of hospital admission for stroke from different hot events by sex, age and categorical for stroke by adjusting the categories of events | 5 |
| **Table S4** | Cumulative risks of stroke from different hot events by sex and age. | 6 |


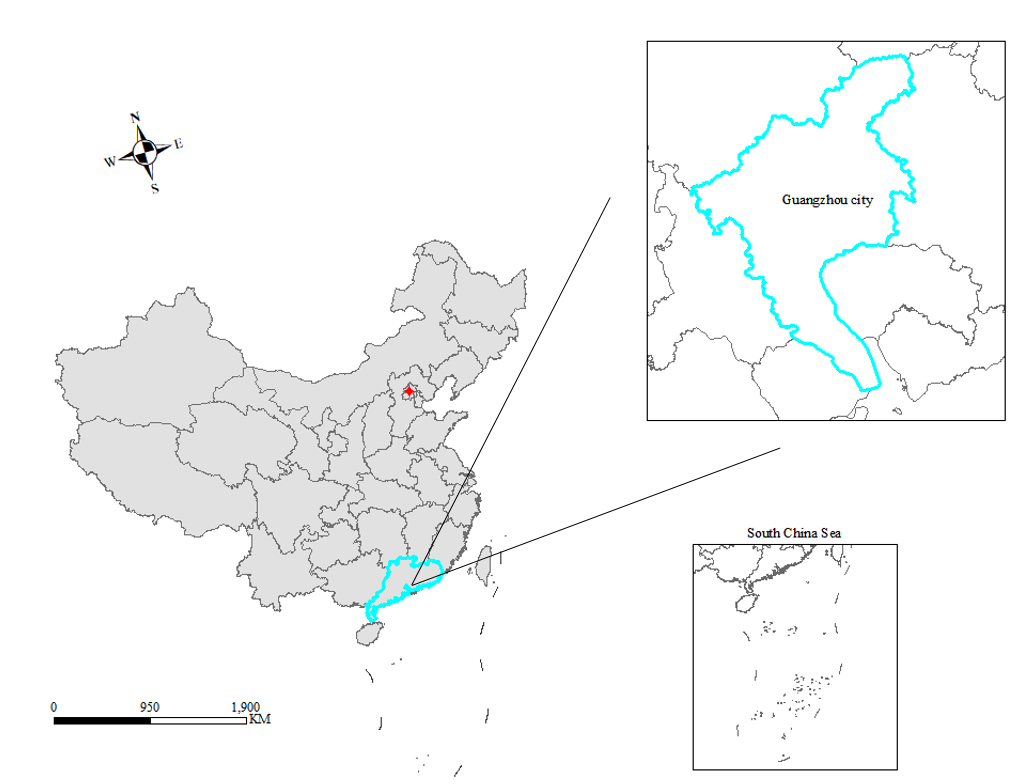


**Figure S1 The location of Guangzhou city in present study**

**Table S1 Cumulative relative risks of hospital admission for stroke from different hot events by sex, age and categorical for stroke by adjusting the maximum lag periods and removing humility**

|  | Hot event & high-severity drought ^a^ | | |  | Hot event & high-severity drought ^b^ | | |  | Hot event & high-severity drought ^c^ | | |
| --- | --- | --- | --- | --- | --- | --- | --- | --- | --- | --- | --- |
|  | RR | 95%CI | |  | RR | 95%CI | |  | RR | 95%CI | |
| All | **1.17** | **1.02** | **1.33** |  | **1.19** | **1.03** | **1.37** |  | **1.19** | **1.02** | **1.39** |
| Types of stroke |  |  |  |  |  |  |  |  |  |  |  |
| SAH | 0.93 | 0.60 | 1.44 |  | 0.92 | 0.57 | 1.47 |  | 1.01 | 0.62 | 1.66 |
| IS | **1.18** | **1.03** | **1.34** |  | **1.19** | **1.04** | **1.38** |  | **1.21** | **1.04** | **1.42** |
| ICH | 1.20 | 0.98 | 1.47 |  | 1.20 | 0.97 | 1.49 |  | 1.15 | 0.91 | 1.46 |
| TIA | 1.10 | 0.85 | 1.43 |  | 1.13 | 0.86 | 1.50 |  | 1.11 | 0.82 | 1.50 |
| OTS | 1.15 | 0.96 | 1.38 |  | 1.19 | 0.98 | 1.44 |  | 1.17 | 0.95 | 1.45 |
| Age |  |  |  |  |  |  |  |  |  |  |  |
| Age≥60 | **1.16** | **1.02** | **1.33** |  | **1.19** | **1.03** | **1.37** |  | **1.20** | **1.02** | **1.40** |
| Age<60 | **1.18** | **1.00** | **1.39** |  | 1.19 | 0.99 | 1.42 |  | 1.17 | 0.96 | 1.42 |
| Gender |  |  |  |  |  |  |  |  |  |  |  |
| Male | **1.17** | **1.02** | **1.34** |  | **1.18** | **1.02** | **1.37** |  | **1.18** | **1.01** | **1.39** |
| Female | **1.16** | **1.00** | **1.35** |  | **1.19** | **1.02** | **1.40** |  | **1.20** | **1.01** | **1.42** |

*Note:*

*The bold values mean statistic significance.*

^a^ Main model was repeated by changing the maximum lag period from 3 to 1 day.

^b^ Main model was repeated by changing the maximum lag period from 3 to 2 day.

^c^ Main model was repeated by removing humidity.

**Table S2 Cumulative relative risks of hospital admission for stroke from different hot events by sex, age and categorical for stroke by adjusting the air pollution**

|  | Hot event & high-severity drought ^a^ | | |  | Hot event & high-severity drought ^b^ | | |  | Hot event & high-severity drought ^c^ | | |
| --- | --- | --- | --- | --- | --- | --- | --- | --- | --- | --- | --- |
|  | RR | 95%CI | |  | RR | 95%CI | |  | RR | 95%CI | |
| All | **1.18** | **1.01** | **1.38** |  | **1.17** | **1.00** | **1.37** |  | **1.17** | **1.00** | **1.37** |
| Types of stroke |  |  |  |  |  |  |  |  |  |  |  |
| SAH | 0.97 | 0.59 | 1.59 |  | 0.98 | 0.59 | 1.61 |  | 0.98 | 0.59 | 1.61 |
| IS | **1.20** | **1.03** | **1.40** |  | **1.19** | **1.02** | **1.39** |  | **1.19** | **1.02** | **1.39** |
| ICH | 1.16 | 0.91 | 1.47 |  | 1.14 | 0.90 | 1.45 |  | 1.15 | 0.90 | 1.45 |
| TIA | 1.06 | 0.78 | 1.44 |  | 1.05 | 0.77 | 1.42 |  | 1.06 | 0.78 | 1.44 |
| OTS | 1.17 | 0.94 | 1.45 |  | 1.16 | 0.93 | 1.43 |  | 1.15 | 0.93 | 1.43 |
| Age |  |  |  |  |  |  |  |  |  |  |  |
| Age≥60 | **1.19** | **1.01** | **1.39** |  | **1.18** | **1.01** | **1.38** |  | **1.17** | **1.00** | **1.37** |
| Age<60 | 1.16 | 0.95 | 1.41 |  | 1.15 | 0.94 | 1.40 |  | 1.15 | 0.94 | 1.40 |
| Gender |  |  |  |  |  |  |  |  |  |  |  |
| Male | **1.18** | **1.00** | **1.38** |  | 1.16 | 0.99 | 1.36 |  | 1.16 | 0.99 | 1.36 |
| Female | **1.19** | **1.00** | **1.41** |  | 1.18 | 0.99 | 1.40 |  | 1.18 | 0.99 | 1.40 |

*Note:*

*The bold values mean statistic significance.*

^a^ Main model was repeated by removing PM_2.5_.

^b^ Main model was repeated by removing PM_2.5_ but including O_3_.

^c^ Main model was repeated by including PM_2.5_ and O_3_.

**Table S3 Cumulative relative risks of hospital admission for stroke from different hot events by sex, age and categorical for stroke by adjusting the categories of events**

|  | Hot event & high-severity drought ^a^ | | |  | Hot event & high-severity drought ^b^ | | |
| --- | --- | --- | --- | --- | --- | --- | --- |
|  | RR | 95%CI | |  | RR | 95%CI | |
| All | **1.18** | **1.01** | **1.38** |  | **1.18** | **1.01** | **1.38** |
| Types of stroke |  |  |  |  |  |  |  |
| SAH | 1.00 | 0.61 | 1.65 |  | 0.96 | 0.58 | 1.59 |
| IS | **1.20** | **1.03** | **1.40** |  | **1.20** | **1.03** | **1.40** |
| ICH | 1.16 | 0.92 | 1.47 |  | 1.17 | 0.92 | 1.48 |
| TIA | 1.06 | 0.78 | 1.43 |  | 1.12 | 0.82 | 1.52 |
| OTS | 1.17 | 0.95 | 1.45 |  | 1.18 | 0.95 | 1.46 |
| Age |  |  |  |  |  |  |  |
| Age≥60 | **1.19** | **1.01** | **1.39** |  | **1.19** | **1.02** | **1.40** |
| Age<60 | 1.16 | 0.95 | 1.41 |  | 1.15 | 0.95 | 1.41 |
| Gender |  |  |  |  |  |  |  |
| Male | **1.17** | **1.00** | **1.37** |  | **1.18** | **1.00** | **1.38** |
| Female | **1.19** | **1.00** | **1.41** |  | **1.19** | **1.00** | **1.42** |

*Note:*

*The bold values mean statistic significance.*

^a^ Events were expanded to four categories: normal temperature, hot event, hot event combined with low-severity drought, and hot event combined with high-severity drought

^b^ Events were expanded to eight categories: normal temperature & neither wet nor drought conditions, independent wet conditions with normal temperature but SPEI > 1, independent drought conditions with normal temperature but SPEI < -1, hot event, hot event with low-severity wet, hot event with high-severity wet, hot event with low-severity drought, and hot event with high-severity drought.

**Table S4 Cumulative risks of stroke from different hot events by sex and age.**

|  | Hot event | | | P Value for Effect Modification |  | Hot event & low-severity drought | | | P Value for Effect Modification |  | Hot event & high-severity drought | | | P Value for Effect Modification |
| --- | --- | --- | --- | --- | --- | --- | --- | --- | --- | --- | --- | --- | --- | --- |
|  | RR | 95%CI | |  |  | RR | 95%CI | |  |  | RR | 95%CI | |  |
| Age |  |  |  |  |  |  |  |  |  |  |  |  |  |  |
| Age≥60 | 1.05 | 0.98 | 1.13 | 0.60 |  | 0.98 | 0.86 | 1.12 | 0.31 |  | **1.19** | **1.01** | **1.39** | 0.85 |
| Age<60 | 1.02 | 0.94 | 1.12 |  |  | 1.09 | 0.93 | 1.27 |  |  | 1.16 | 0.95 | 1.41 |  |
| Gender |  |  |  |  |  |  |  |  |  |  |  |  |  |  |
| Male | 1.04 | 0.96 | 1.12 | 0.74 |  | 1.00 | 0.87 | 1.14 | 0.92 |  | **1.17** | **1.00** | **1.38** | 0.89 |
| Female | 1.06 | 0.98 | 1.14 |  |  | 1.01 | 0.87 | 1.16 |  |  | **1.19** | **1.00** | **1.41** |  |
